# 5-Demethylnobiletin Inhibits Cell Proliferation, Downregulates ID1 Expression, Modulates the NF-κB/TNF-α Pathway and Exerts Antileukemic Effects in AML Cells

**DOI:** 10.3390/ijms23137392

**Published:** 2022-07-02

**Authors:** Pei-Yi Chen, Chih-Yang Wang, En-Ci Tsao, Yu-Ting Chen, Ming-Jiuan Wu, Chi-Tang Ho, Jui-Hung Yen

**Affiliations:** 1 Center of Medical Genetics, Hualien Tzu Chi Hospital, Buddhist Tzu Chi Medical Foundation, Hualien 97004, Taiwan; pyc571@gmail.com; 2 Department of Molecular Biology and Human Genetics, Tzu Chi University, Hualien 97004, Taiwan; kiki841227@gmail.com (E.-C.T.); b7291537@gmail.com (Y.-T.C.); 3 Ph.D. Program for Cancer Molecular Biology and Drug Discovery, Taipei Medical University, Taipei 11031, Taiwan; chihyang@tmu.edu.tw; 4 Graduate Institute of Cancer Biology and Drug Discovery, Taipei Medical University, Taipei 11031, Taiwan; 5 Department of Biotechnology, Chia Nan University of Pharmacy and Science, Tainan 71710, Taiwan; mingjiuanwu@gmail.com; 6 Department of Food Science, Rutgers University, New Brunswick, NJ 08901, USA; ctho@sebs.rutgers.edu; 7 Institute of Medical Sciences, Tzu Chi University, Hualien 970, Taiwan

**Keywords:** AML, 5-demethylnobiletin, GSEA, ID1, NF-κB, cytarabine

## Abstract

Acute myeloid leukemia (AML) is characterized by the dysregulation of hematopoietic cell proliferation, resulting in the accumulation of immature myeloid cells in bone marrow. 5-Demethylnobiletin (5-demethyl NOB), a citrus 5-hydroxylated polymethoxyflavone, has been reported to exhibit various bioactivities, such as antioxidant, anti-inflammatory and anticancer properties. In this study, we investigated the antileukemic effects of 5-demethyl NOB and its underlying molecular mechanisms in human AML cells. We found that 5-demethyl NOB (20–80 μM) significantly reduced human leukemia cell viability, and the following trend of effectiveness was observed: THP-1 ≈ U-937 > HEL > HL-60 > K562 cells. 5-Demethyl NOB (20 and 40 μM) modulated the cell cycle through the regulation of p21, cyclin E1 and cyclin A1 expression and induced S phase arrest. 5-Demethyl NOB also promoted leukemia cell apoptosis and differentiation. Microarray-based transcriptome, Gene Ontology (GO) and Gene Set Enrichment Analysis (GSEA) of differentially expressed genes (DEGs) analysis showed that the expression of inhibitor of differentiation/DNA binding 1 (ID1), a gene associated with the GO biological process (BP) cell population proliferation (GO: 0008283), was most strongly suppressed by 5-demethyl NOB (40 μM) in THP-1 cells. We further demonstrated that 5-demethyl NOB-induced ID1 reduction was associated with the inhibition of leukemia cell growth. Moreover, DEGs involved in the hallmark gene set NF-κB/TNF-α signaling pathway were markedly enriched and downregulated by 5-demethyl NOB. Finally, we demonstrated that 5-demethyl NOB (20 and 40 μM), combined with cytarabine, synergistically reduced THP-1 and U-937 cell viability. Our current findings support that 5-demethyl NOB dramatically suppresses leukemia cell proliferation and may serve as a potential phytochemical for human AML chemotherapy.

## 1. Introduction

Acute myeloid leukemia (AML) is an aggressive disease characterized by the transformation of hematopoietic stem cells or precursor cells into malignant leukemia cells, and the process is driven by several acquired chromosomal and genetic abnormalities. These recurrent genetic aberrations are the most essential factors that enhance the proliferative activity of leukemia cells and impede their ability to undergo hematopoietic differentiation or apoptosis [1]. Despite great research efforts in understanding the pathogenesis of the disease, the standard therapy for AML remains unchanged and has consisted of intensive chemotherapy with cytarabine plus anthracycline for several decades [2]. Indeed, the long-term survival of patients is not optimistic due to primary resistance or clonal evolution leading to adaptive resistance, and up to 70% of adults and 30% of children will not survive beyond 5 years after the initial clinical response due to relapsing disease [3,4]. Recent studies using single-cell technologies illustrated the complex situation of multiple resistant subclones coexisting and evolving during therapy and acting as a barrier to the long-term success of targeted therapies [5,6]. An increase in understanding the molecular pathogenesis of AML drives the development of novel agents in the prevention or treatment of AML; in particular, small molecules, such as tyrosine kinase inhibitors (TKIs) and monoclonal antibodies, that target cancer cells to modulate signaling molecules are currently being investigated in clinical trials [7].

Comprehensive studies have reported that cytogenetic and molecular abnormalities in AML lead to the production of aberrant oncoproteins, tumor suppressors, transcription factors, regulators of apoptosis and differentiation and signaling molecules, which promote cancer cell proliferation, survival and leukemia development [8]. In addition to implications of genetic origin, recent studies indicate that the inflammatory cytokine signaling network in the cancer microenvironment may contribute to the development of AML, independent of genetic mutation status. The dysregulation of the expression of pro- and anti-inflammatory mediators, such as tumor necrosis factor-α (TNF-α) and interleukins, may significantly promote leukemia cell growth and survival in AML [9,10]. Therefore, investigations into targeting or regulating signaling molecules or pathways relevant to cell proliferation, survival, apoptosis and the inflammatory response represent a novel and promising approach to the prevention and treatment of AML patients. 

Several natural antioxidant phytochemicals, such as flavonoids, possess anti-inflammatory activity and are essential sources for developing preventive or therapeutic agents for cancers, including hematopoietic malignancies [11,12,13]. Citrus fruit and its flavonoid phytochemicals isolated from peel, such as polymethoxyflavones (PMFs) and hydroxylated PMFs, have been identified as compounds with a high potential for health promotion and cancer prevention or treatment [14]. In our previous reports, nobiletin (NOB), a citrus PMF, displayed strong antileukemic effects and promoted myeloid differentiation not only in AML but also in CML cells [15,16]. Several natural hydroxylated derivatives of NOB have also been documented to have potential for chemoprevention or chemotherapy of cancers [17,18]. One of the promising NOB derivatives is 5-demethylnobiletin (5-hydroxy-6,7,8,3′,4′-pentamethoxyflavone, 5-demethyl NOB), the most abundant demethylated PMF that is mainly formed through NOB autohydrolysis in the long-term storage of citrus peels and has been reported to exhibit several biological activities, including anti-inflammatory, anti-atherogenic, neuroprotective, melanogenic and anticancer properties [19,20,21,22,23,24]. Several in vitro and in vivo studies have demonstrated that 5-demethyl NOB prevents inflammation-associated carcinogenesis; induces cell cycle arrest, autophagy and apoptosis; and suppresses cell proliferation by modulating key signaling proteins and pathways in various types of solid tumors, including colon cancer, lung cancer, and gastric cancer [25,26,27,28]. In a study on leukemia, Li et al., demonstrated that 5-demethyl NOB inhibited cell proliferation and induced apoptosis in a human HL-60 cell line [29]. However, there is no report to date that 5-demethyl NOB modulates key regulators or signaling pathways related to cell proliferation to specifically inhibit leukemia cell growth. In this study, we aimed to investigate the antileukemic effects of 5-demethyl NOB and the underlying molecular mechanisms in human AML cell lines. We also investigated the effect of cytarabine (Ara-C) combined with 5-demethyl NOB to reduce the viability of human AML cells.

## 2. Results 

### 2.1. Effects of 5-Demethyl NOB on Human Leukemia Cell Viability

**Figure 1:**
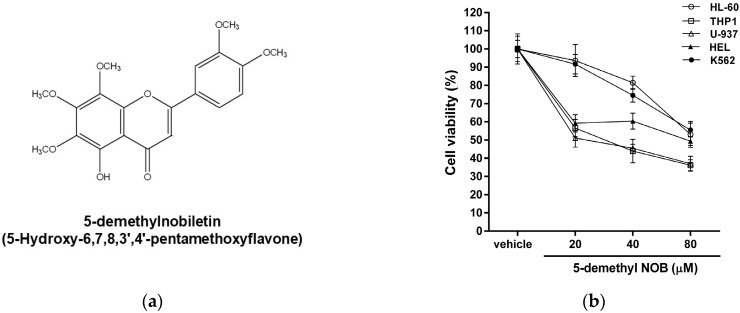

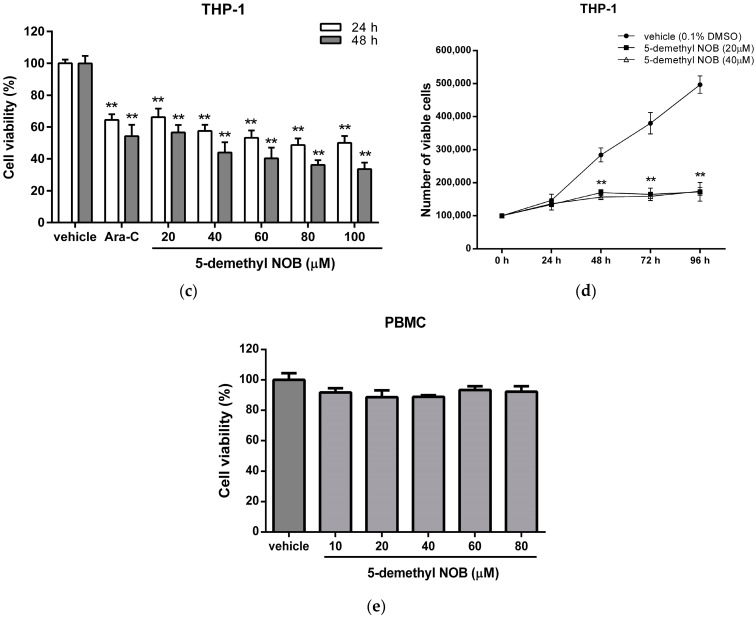
The effect of 5-demethyl NOB on leukemia cell viability. (**a**) The chemical structure of 5-demethylnobiletin (5-hydroxy-6,7,8,3′,4′-pentamethoxyflavone). (**b**) HL-60, THP-1, U937, HEL and K562 cells were treated with vehicle (0.1% DMSO) or 5-demethyl NOB (20–80 μM) for 48 h. Cell viability was measured by MTT assay. (**c**) THP-1 cells were treated with vehicle, cytarabine (Ara-C, 20 μM) or 5-demethyl NOB (20–100 μM) for 24 and 48 h. Cell viability was measured by MTT assay. (**d**) THP-1 cells (2.5 × 10^5^/mL) were seeded and incubated with 5-demethyl NOB (20 and 40 μM) for 0–96 h. The number of cells was measured by counting viable cells using trypan blue staining. (**e**) PBMCs were treated with 5-demethyl NOB (10–80 μM) for 48 h, and cell viability was measured by MTT assay. The data represent the mean ± SD of three independent experiments. ** *p* < 0.01 represents significant differences compared to the vehicle-treated group.

Citrus PMFs have been reported to suppress cancer cell growth in several types of tumors, including leukemia [30]. Here, we first examined the effect of a citrus PMF 5-demethyl NOB (Figure 1a) on leukemia cell viability. Five human leukemia cell lines, including AML cells (HL-60, THP-1, U-937, and HEL cells) and a CML cell line (K562 cells), were treated with vehicle (0.1% DMSO) or 5-demethyl NOB (20, 40 and 80 μM) for 48 h, and cell viability was measured by MTT assay. As shown in Figure 1b, human leukemia cells treated with 5-demethyl NOB showed significantly decreased viability. The IC_50_ values (inhibitory concentration of 50% cell growth) in 5-demethyl NOB-treated HL-60, THP-1, U-937, HEL, and K562 cells were 85.7 μM, 32.3 μM, 30.4 μM, 65.3 μM, and 91.5 μM, respectively. These data suggested that the effectiveness of 5-demethyl NOB against leukemia cells exhibited the following trend: THP-1≈U-937 > HEL > HL-60 > K562. These data indicated that 5-demethyl NOB displays significant and specific cytotoxic effects on human leukemia cells. These results also suggested that monocytic AML cells, such as THP1 and U-937 cells, seem to be more sensitive to 5-demethyl NOB than other leukemia cells.

We further examined the effect of 5-demethyl NOB on THP-1 cell viability. THP-1 cells were treated with vehicle, cytarabine (Ara-C, as a positive control) and 5-demethyl NOB (20−100 μM) for 24 and 48 h; then, cell viability was determined by MTT assay. As shown in Figure 1c, the viability in THP-1 cells treated with Ara-C (20 μM) for 24 h and 48 h was reduced to 64.4 ± 3.6% and 54.3 ± 7.1%, as compared with the vehicle-treated group (100.0 ± 2.3% and 100.0 ± 4.7%), respectively (*p* < 0.01). The viability of cells treated with 5-demethyl NOB (20, 40, 80 and 100 μM) for 24 h decreased to 69.24 ± 6.5%, 57.9 ± 3.5%, 51.2 ± 4.6%, 48.7 ± 4.6% and 50.0 ± 4.4% of vehicle control, respectively, whereas the cell viability after 48 h treatment decreased to 56.1 ± 4.2%, 44.9 ± 6.2%, 40.3 ± 6.8%, 35.2 ± 3.0% and 33.4 ± 4.5% of vehicle control, respectively (*p* < 0.01). Moreover, to examine whether 5-demethyl NOB affects leukemia cell growth, viable THP-1 cells (2 × 10^5^/mL at seeding) were treated with vehicle or 5-demethyl NOB (20 and 40 μM) for 24–96 h, and the numbers of viable cells were measured by counting the cells. As shown in Figure 1d, the number of viable cells not increased, and cell proliferation was significantly inhibited in 5-demethyl NOB-treated cells for 48–96 h compared to the 0 h group. Moreover, the viability of 5-demethyl NOB (10–80 μM)-treated peripheral blood mononuclear cells (PBMCs) was also determined, and the data showed that no significant cytotoxicity was detected in PBMCs (Figure 1e). These data supported that 5-demethyl NOB significantly decreased the viability of different human AML cells; however, no cytotoxicity in compound-treated normal PBMCs was detected.

### 2.2. Effects of 5-Demethyl NOB on Cell Cycle Progression in THP-1 Cells

**Figure 2:**
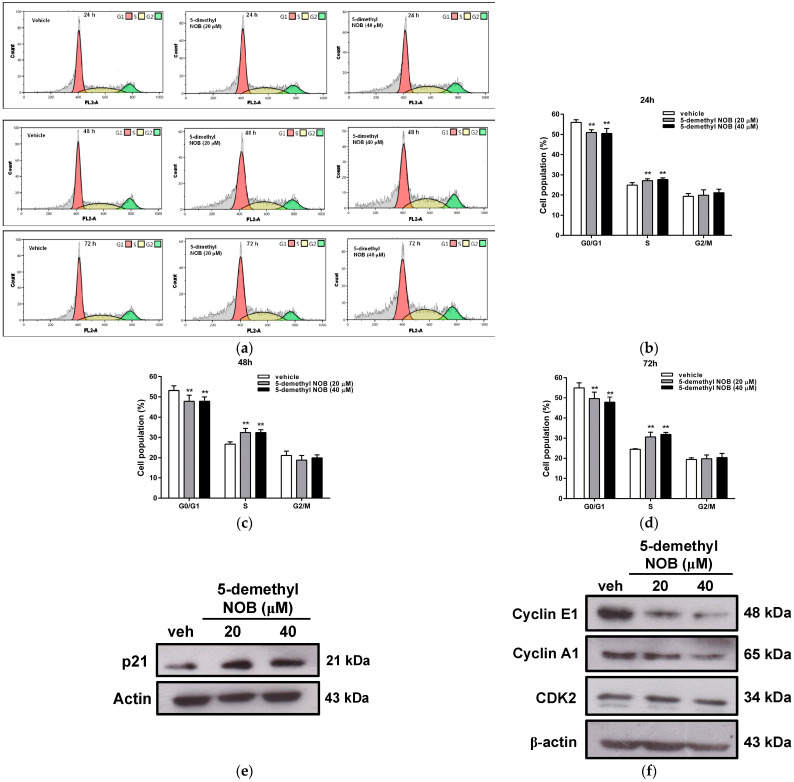
Effects of 5-demethyl NOB on cell cycle distribution and regulatory gene expression. THP-1 cells were treated with vehicle or 5-demethyl NOB (20 and 40 μM) for 24–72 h, and the cell cycle distribution was detected by flow cytometric analysis. (**a**) A representative histogram of cell cycle distribution. THP-1 cells were treated with 5-demethyl NOB (20 and 40 μM) for (**b**) 24 h, (**c**) 48 h, and (**d**) 72 h, and the cell populations in G0/G1, S and G2/M phases were quantified. The data represent the mean ± SD of three independent experiments. ** *p* < 0.01 represents significant differences compared to the vehicle-treated group. Western blot analysis of (**e**) p21, (**f**) cyclin E1, cyclin A1, CDK2 and actin expression in THP-1 cells treated with vehicle or 5-demethyl NOB (20 and 40 μM) for 48 h.

We next investigated the effect of 5-demethyl NOB on cell cycle progression in THP-1 cells using flow cytometry analysis. As shown in Figure 2a–d, the incubation of THP-1 cells with 5-demethyl NOB (20 and 40 μM) for 24–72 h resulted in a significant decrease in the cell population in the G1 phase and accumulation in the S phase compared to vehicle (0.1% DMSO)-treated cells. Treatment with 5-demethyl NOB did not change the cell distribution in the G2/M phase. These data indicated that 5-demethyl NOB could induce cell cycle arrest in S phase to impede THP-1 cell proliferation. 

Furthermore, we examined the effect of 5-demethyl NOB on the cell cycle regulators involved in controlling the G1/S phase transition and S phase arrest. As shown in Figure 2e,f, the level of the CDK inhibitor p21^Waf1/Cip1^ (p21) protein was significantly increased, whereas cyclin E1 and cyclin A1 proteins were reduced in 5-demethyl NOB-treated THP-1 cells. The protein level of CDK2 was not significantly altered by 5-demethyl NOB in THP-1 cells. These data revealed that 5-demethyl NOB could induce cell cycle arrest and inhibit cell growth in human AML cells.

### 2.3. 5-Demethyl NOB Induced Cell Apoptosis and Differentiation in AML Cell Lines

**Figure 3:**
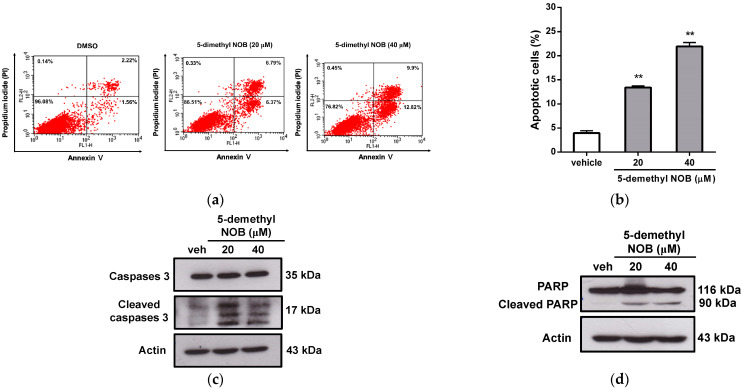

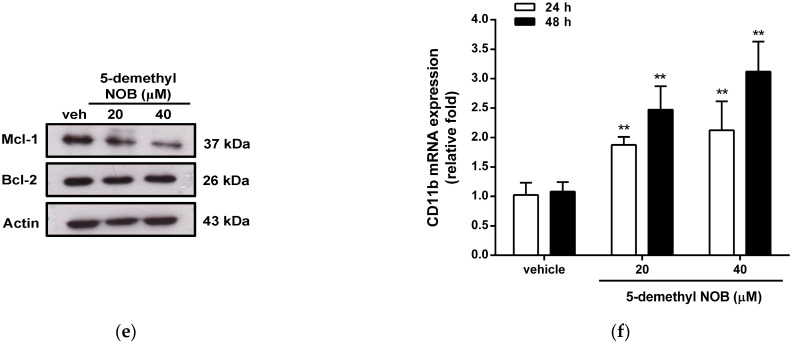
Effects of 5-demethyl NOB on cell apoptosis and differentiation. THP-1 cells were treated with vehicle or 5-demethyl NOB (20 and 40 μM) for 48 h, and cell apoptosis was detected by flow cytometric analysis. (**a**) A representative histogram of cell apoptosis. (**b**) The percentage of apoptotic cells was quantified. (**c**–**e**) Western blot analysis of apoptosis-related gene expression after 5-demethyl NOB treatment for 48 h. (**f**) THP-1 cells were treated with vehicle or 5-demethyl NOB (20 and 40 μM) for 24 h and 48 h. CD11b mRNA expression was measured by RT–qPCR analysis. The data represent the mean ± SD of three independent experiments. ** *p* < 0.01 represents significant differences compared to the vehicle-treated group.

Given that 5-demethyl NOB modulates cell cycle progression, we further investigated whether 5-demethyl NOB could affect THP-1 cell apoptosis and differentiation. To examine whether cell apoptosis could be induced by 5-demethyl NOB in THP-1 cells, we analyzed the apoptotic effect of compound-treated leukemia cells using flow cytometric analysis. As shown in Figure 3a,b, 5-demethyl NOB (20 and 40 μM) significantly caused an increase in apoptotic cell populations (approximately 13% and 22%, respectively) compared to the vehicle-treated group (approximately 3.5%) (*p* < 0.01). We further analyzed apoptosis-related proteins using Western blot analysis. As shown in Figure 3c,d, treatment of THP-1 cells with 5-demethyl NOB markedly elevated the levels of cleaved caspase 3 and poly (ADP-ribose) polymerase 1 (PARP1) proteins, which are hallmarks of apoptosis. Moreover, 5-demethyl NOB also reduced the level of the antiapoptotic protein Mcl-1 in THP-1 cells (Figure 3e). These results indicated that 5-demethyl NOB could significantly promote cell apoptosis in THP-1 cells. Moreover, we examined the effect of 5-demethyl NOB on cell differentiation by detecting the mRNA expression of the CD11b gene, a monocytic differentiation marker, in THP-1 cells. Figure 3f illustrates that THP-1 cells treated with 5-demethyl NOB (20 and 40 μM) for 24–48 h markedly increased CD11b mRNA expression compared with that in the vehicle-treated group, respectively (*p* < 0.01). Similar effects on the stimulation of cell differentiation were also found in 5-demethyl NOB-treated U-937 and HL-60 cells compared to vehicle-treated cells (Figure S1). These data indicated that 5-demethyl NOB significantly promoted myeloid leukemia cell differentiation. These results show that 5-demethyl NOB promote cell apoptosis and differentiation in AML cells.

### 2.4. Analysis of 5-Demethyl NOB-Modulated Gene Expression in AML Cells

**Figure 4:**
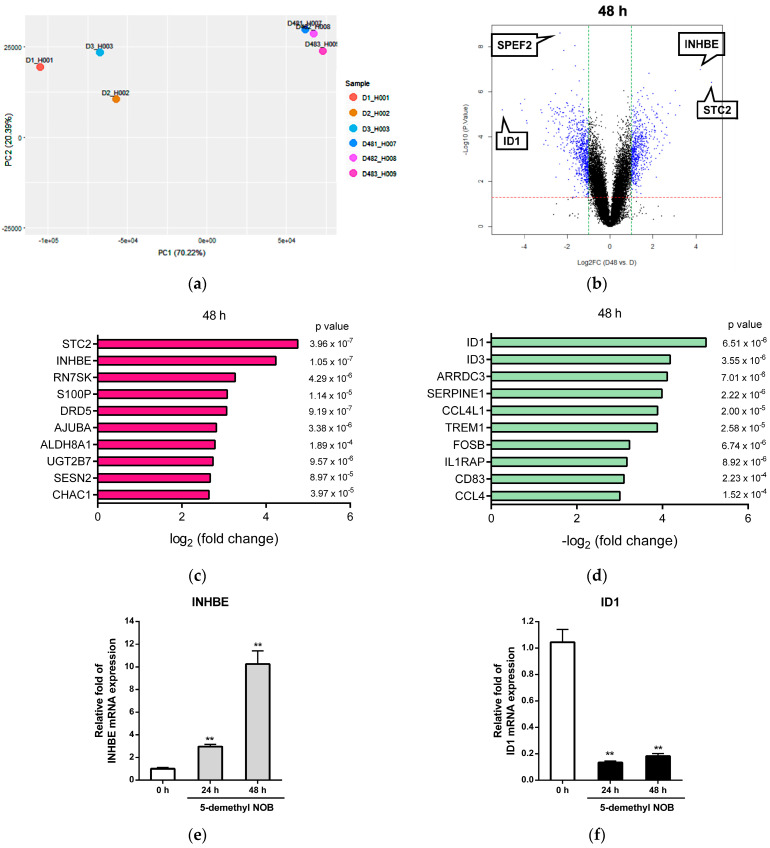
Microarray analysis of 5-demethyl NOB-treated THP-1 cells. THP-1 cells were treated with vehicle or 5-demethyl NOB (40 μM) for 48 h, and the mRNA expression profiles were determined by human cDNA microarray. (**a**) Principal component analysis (PCA) of microarray data obtained from analyzing triplicate samples of vehicle (H001-H003) and 5-demethyl NOB (H007-H009) treatment. (**b**) Volcano plots of total gene expression profiles of the 5-demethyl NOB-treated cells. Each dot represents the mean expression (*n* = 3) of the individual gene obtained from a normalized dataset. Genes above the cutoff values (the red and green dotted lines) were considered differentially expressed genes (DEGs) (blue dots). (**c**) The top 10 significantly upregulated and (**d**) downregulated genes in response to 5-demethyl NOB treatment. RT-qPCR analysis of (**e**) INHBE and (**f**) ID1 mRNA expression in 5-demethyl NOB-treated cells for 24 and 48 h. The data represent the mean ± SD of three independent experiments. ** *p* < 0.01 represents significant differences compared to the 0 h group.

The results shown above indicated that 5-demethyl NOB inhibits cell growth and induces cell apoptosis as well as differentiation to prevent cancer cell proliferation in AML cells. To investigate the potential genes involved in the antiproliferative effects of 5-demethyl NOB in AML cells and explore the molecular mechanisms, we examined the DEGs of compound-treated leukemia cells using a human genome-wide microarray system. THP-1 cells were treated with 5-demethyl NOB (40 μM) for 48 h, and the transcriptomic profiles were analyzed in triplicate using a human cDNA microarray. The quality control of the sample level was measured using principal component analysis (PCA), and the data showed that three replicates of each treatment were closely clustered (Figure 4a). The selected DEGs were based on log_2_ [fold change (FC)] ≥1.0 (upregulation) or log_2_ [FC] ≤ −1.0 (downregulation), and *p* < 0.05. In the 5-demethyl NOB-treated group, there were 552 upregulated and 694 downregulated genes in cells treated for 48 h compared to the 0 h control group (Table 1).

Figure 4b illustrates the general transcriptome changes as volcano plots in 5-demethyl NOB treatment for 48 h. Figure 4c,d show the top 10 significantly upregulated and downregulated DEGs in 5-demethyl NOB-treated cells, respectively. The two most significantly upregulated genes induced by 5-demethyl NOB are stanniolcalcin 2 (STC2), a secreted glycoprotein that plays a role in the regulation of cellular calcium/phosphate homeostasis [31]; and inhibin subunit beta E (INHBE), a member of the transforming growth factor-β (TGF-β) family of proteins regulating cell proliferation, apoptosis, and immune response [32,33]. The most significantly downregulated gene is ID1, which encodes an inhibitor of differentiation/DNA binding 1 protein that can form heterodimers with members of the basic helix–loop–helix (HLH) family proteins but has no DNA binding activity [34,35]. ID1 is an oncogene that promotes cancer cell proliferation, survival and tumorigenesis in several types of cancer [36]. The differential expression of INHBE and ID1 transcription in 5-demethyl NOB-treated cells was verified, and the data are shown in Figure 4e,f. 

### 2.5. Analysis of 5-Demethyl NOB-Regulated Biological Processes (BPs)

**Figure 5:**
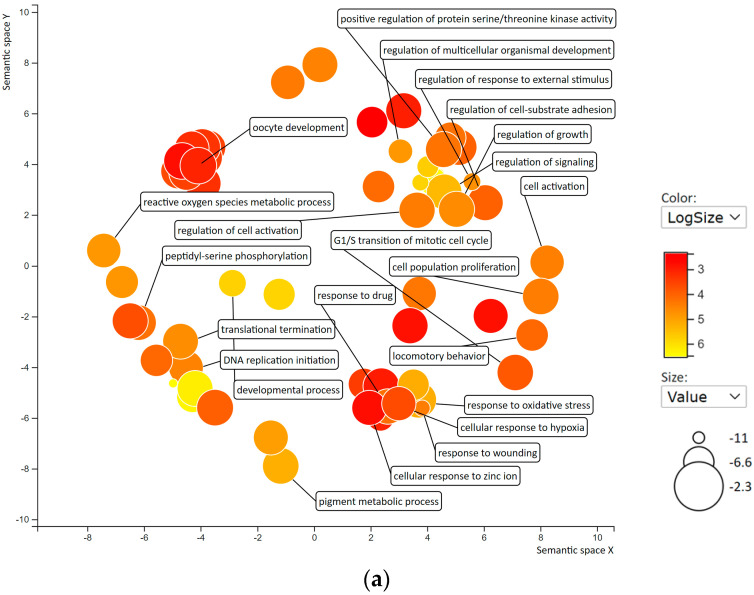

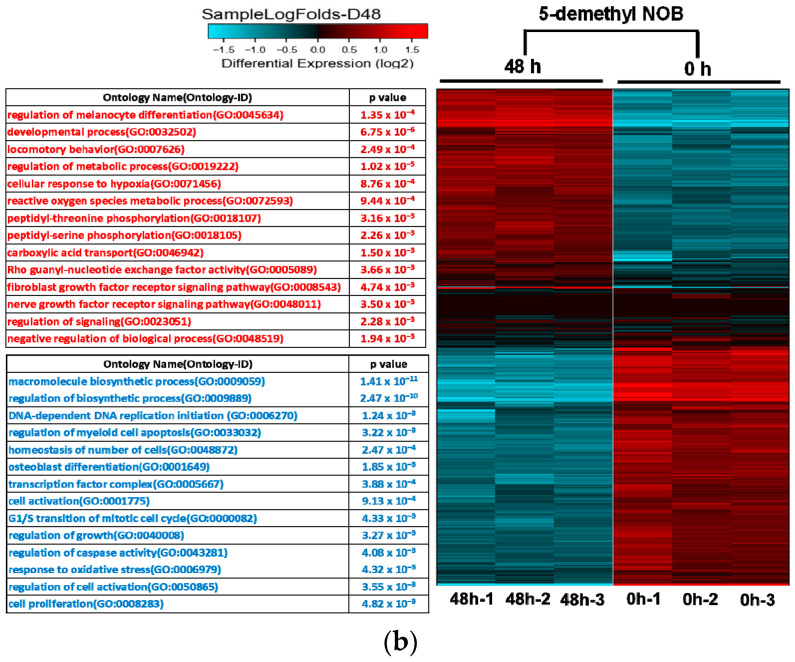
Enrichment analysis and visualization of Gene Ontology (GO) results for DEGs in response to 5-demethyl NOB treatment. (**a**) Significantly enriched GO biological processes (BPs) in THP-1 cells treated with vehicle vs. 5-demethyl NOB (40 μM) were analyzed by REVIGO. The scatterplot represents functional clusters. The bubble color and size represent the *p* values and the frequency of the GO BP in the database, respectively. (**b**) Heatmap of enriched GO BP terms in response to 5-demethyl NOB (40 μM) treatment. GO processes listed in the upper and lower panels are terms of upregulated and downregulated DEGs, respectively. Only some of the significantly representative GO terms are shown.

To analyze Gene Ontology (GO) terms regulated by 5-demethyl NOB in THP-1 cells, the set of significant DEGs was subjected to functional annotation for enrichment analysis using the GO database to identify the biological processes (BPs) in which they are involved. The GO BP groups that fulfilled the following criteria, namely DEGs with fold change values greater than 0.5 or less than −0.5 and *p* < 0.05, were included in the “Enrichment GO BP” gene sets using Gene Set Enrichment Analysis (GSEA) from the Molecular Signatures Database (MSigDB) [37]. We further used Reduce plus Visualize Gene Ontology (REVIGO) tool to visualize nonredundant GO annotations on the basis of semantic similarities of GO terms [38] and the Z score-elite to perform GO pruning for overrepresentation analysis (ORA) to determine whether known BPs are enriched in DEGs [39,40] (Tables S1–S3). As shown in Figure 5a, GO BPs associated with the modulation of cancer cell progression, such as cell population proliferation, cell differentiation, cell death, regulation of apoptotic process, and cell–cell signaling, were significantly altered by 5-demethyl NOB. Moreover, Figure 5b shows the ORA results indicating that BPs, such as melanocyte differentiation, hypoxia, and signal transduction, were associated with upregulated DEGs in 5-demethyl NOB-treated cells. On the other hand, the regulation of cell proliferation, cell growth and cell cycle G1/S transition were significantly associated with the downregulation of DEGs in 5-demethyl NOB-treated cells. These data were in good agreement with our experimental results indicating that 5-demethyl NOB inhibits cell growth, modulates the cell cycle, induces cell apoptosis, and promotes myeloid differentiation. 

### 2.6. Analysis of Gene Set Enrichment in 5-Demethyl NOB-Treated THP-1 Cells

**Figure 6:**
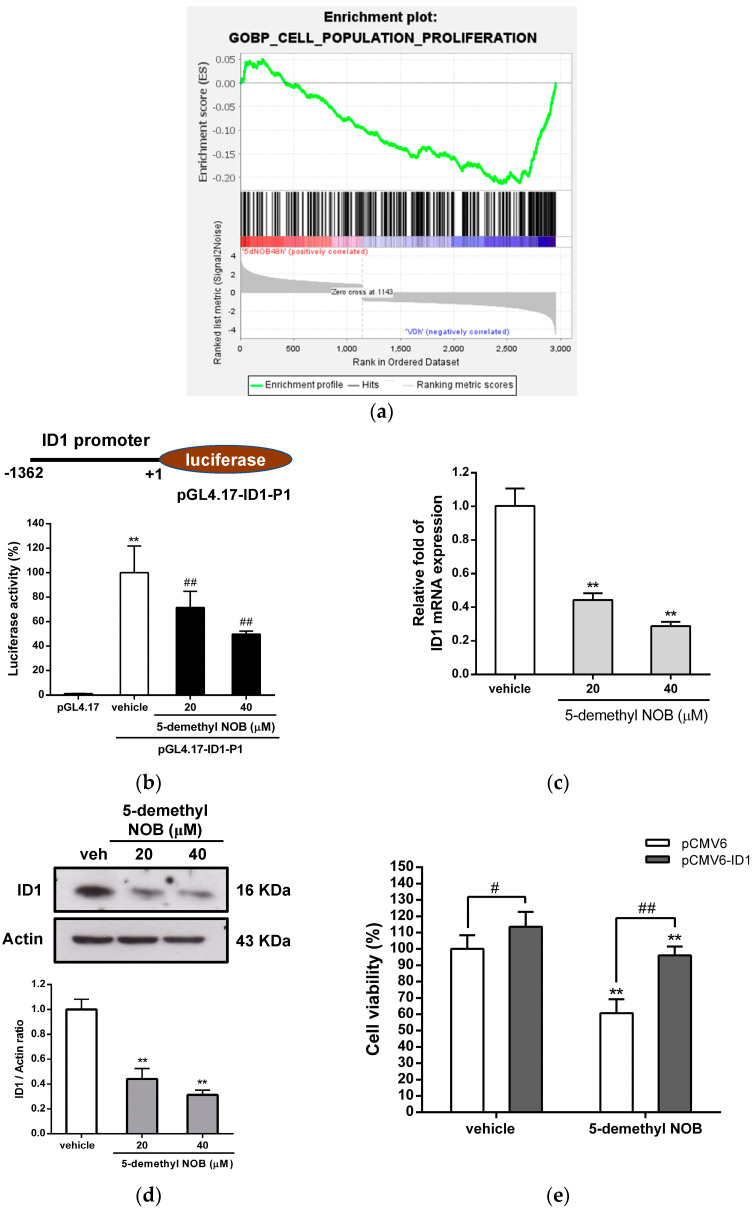
Gene set enrichment analysis (GSEA) and ID1 gene expression in 5-demethyl NOB-treated THP-1 cells. (**a**) GSEA demonstrates that the GO BP signature “Enrichment plot: GO BP Cell Population Proliferation” (GO: 0008283) gene set is enriched with the DEGs of 5-demethyl NOB-treated cells. The barcode plot shows the position of the genes in the gene set. (**b**) The pGL4.17 vector or *ID1* promoter-luciferase plasmid (pGL4.17-ID1-P1) and the *Renilla* luciferase control plasmid were co-transfected into THP-1 cells for 24 h. These cells were then treated with vehicle or 5-demethyl NOB (20 and 40 μM) for 24 h. Luciferase activity was measured and normalized to the *Renilla* control. The data represent the mean ± SD of three independent experiments. ** *p* < 0.01 represents significant differences compared to the control vector-transfected group. ## *p* < 0.01 represents significant differences compared to the vehicle group. (**c**) THP-1 cells were treated with vehicle or 5-demethyl NOB (20 and 40 μM) for 48 h, and ID1 mRNA levels were determined by RT–qPCR analysis. (**d**) Western blot analysis of ID1 and actin proteins. The experiments were performed in triplicate, and a representative blot is shown. The intensity of ID1 versus actin protein was normalized. The data represent the mean ± SD of three independent experiments. ** *p* < 0.01 represents significant differences compared to the vehicle group. (**e**) The pCMV6 control vector or pCMV6-ID1 expression plasmid was transfected into THP-1 cells for 24 h followed by treatment with vehicle or 5-demethyl NOB (40 μM) for 48 h. Cell viability was measured by MTT assay. The viability of the vehicle-treated group (pCMV6-transfected cells) was expressed as 100%. # *p* < 0.05 and ## *p* < 0.01 represent a significant difference compared to the 5-demethyl NOB-treated pCMV6-transfected group.

Furthermore, we used GSEA to explore which gene sets are significantly enriched in 5-demethyl NOB-treated AML cells. The gene sets that were markedly enriched at a false discovery rate (FDR) < 0.25 for 48 h of treatment with 5-demethyl NOB were analyzed. We focused on the “GO BP Cell population proliferation” (GO: 0008283) and identified transcriptional changes involved in this GO term by GSEA [Enrichment score (ES) = −0.2132, normalized ES (NES) = −1.9355, FDR q-value < 0.001, leading edge: tags = 25%, list = 17%, signal = 27%]. An enrichment plot showed that a gene set with 72 core enrichment genes associated with GO: 0008283 was significantly downregulated by 5-demethyl NOB treatment. Among these DEGs, ID1 was the most downregulated gene and is considered a critical regulator involved in the modulation of cell proliferation in 5-demethyl NOB-treated cells (Figure 6a and Table S4). As shown in Figure 6b–d, 5-demethyl NOB (20 and 40 μM) significantly reduced the transcriptional activity of the ID1 promoter and the levels of ID1 mRNA and protein expression in THP-1 cells. Then, we investigated whether the suppression of cell growth by 5-demethyl NOB is associated with the downregulation of ID1 expression. The cells were transfected with the control vector (pCMV6) or ID1 expression plasmid (pCMV6-ID1) followed by treatment with vehicle or 5-demethyl NOB. As shown in Figure 6e, in vehicle- or 5-demethyl NOB-treated THP-1 cells, ID1 overexpression significantly increased the number of viable cells compared with that in control plasmid-transfected cells (*p* < 0.05). ID1 overexpression markedly promoted cell growth and rescued the viability of 5-demethyl NOB-treated cells (*p* < 0.01). These data indicated that ID1 attenuated the antiproliferative effect of 5-demethyl NOB in THP-1 cells. Our data revealed that 5-demethyl NOB-induced ID1 downregulation was associated with the inhibition of AML cell growth.

**Figure 7:**
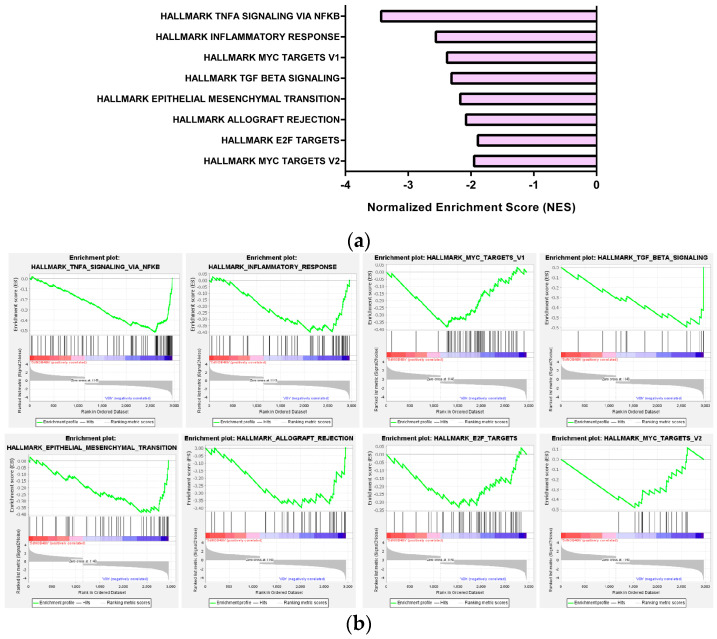

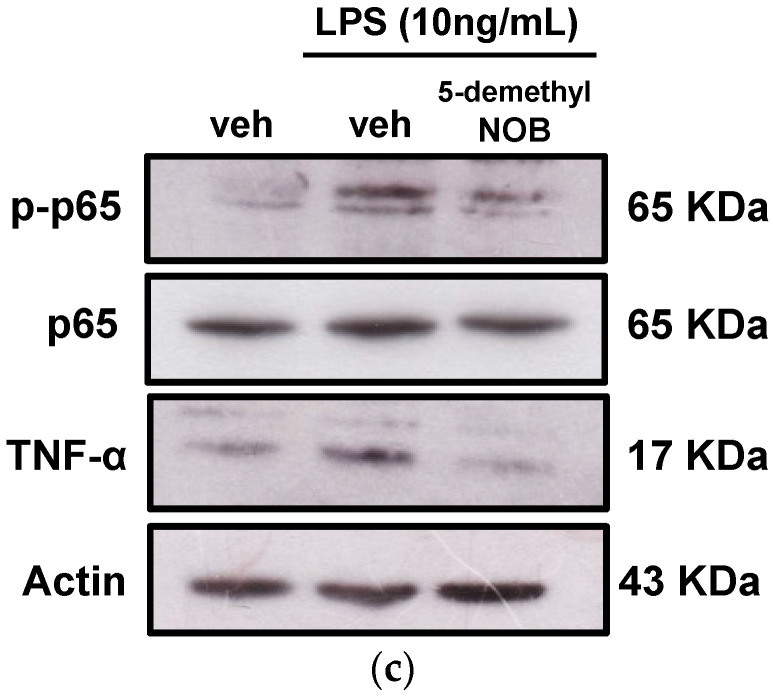
The hallmark gene sets of molecular pathways in response to 5-demethyl NOB treatment. (**a**) The top eight hallmark gene sets of molecular pathways associated with genes downregulated by 5-demethyl NOB. (**b**) GSEA demonstrates that the signature “Hallmark” gene set is enriched in the DEGs by 5-demethyl NOB treatment. (**c**) THP-1 cells were pretreated for 1 h with vehicle or 5-demethyl NOB and then incubated with LPS (10 ng/mL) for 24 h. p-p65, p65, TNF-α and actin proteins were detected by Western blot analysis. A representative blot is shown.

The “Hallmark” gene sets were also analyzed in 5-demethyl NOB-treated cells by GSEA. It was found that the hallmark gene sets of molecular pathways, including TNFα signaling via NF-κB, inflammatory response, MYC targets V1 and V2, TGF beta signaling, epithelial mesenchymal transition, allograft rejection, and E2F targets, were markedly enriched and downregulated by 5-demethyl NOB (NES ≤ −1.7, *p* < 0.01, FDR q < 0.25) (Figure 7a,b and Table S5). The gene sets involved in the signature “Hallmark TNFα signaling via NF-κB” and “Hallmark inflammatory response” showed the most significant downregulation and highest enrichment. Constitutive NF-κB signaling activation and higher TNF-α secretion have been detected in AML, and its aberrant activity is involved in leukemia cells escaping apoptosis and accelerating proliferation [41,42]. Figure 7c shows that 5-demethyl NOB significantly reduced the levels of phosphorylated NF-κB (p-p65) and TNF-α proteins in LPS-treated THP-1 cells. These results supported that the inhibition of the NF-κB activation and TNF-α production by 5-demethyl NOB may play a critical role in the anti-proliferative effect of AML cells.

### 2.7. Effects of Combined Treatment with 5-Demethyl NOB and Cytarabine in AML Cells

**Figure 8:**
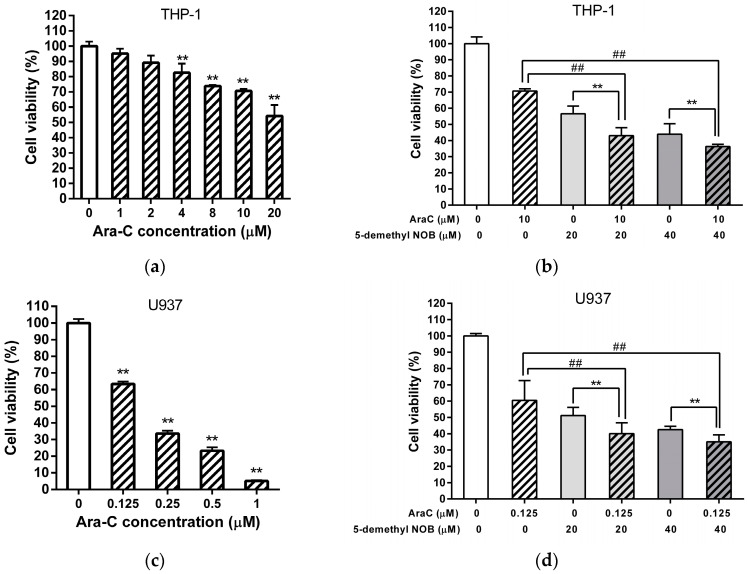
Combination treatment of cytarabine (Ara C) and 5-demethyl NOB in THP-1 and U-937 cells. (**a**) THP-1 cells were treated with Ara C (0–20 μM) for 48 h. Cell viability was measured by MTT assay. The data represent the mean ± SD of three independent experiments. (**b**) THP-1 cells were pretreated with Ara C (10 μM) for 12 h followed by incubation with vehicle or 5-demethyl NOB (20 or 40 μM) for an additional 36 h. Cell viability was measured using the MTT assay. (**c**) U-937 cells were treated with Ara C (0–1 μM) for 48 h. Cell viability was measured by MTT assay. The data represent the mean ± SD of three independent experiments. (**d**) THP-1 cells were pretreated with Ara C (0.125 μM) for 12 h followed by incubation with vehicle or 5-demethyl NOB (20 or 40 μM) for an additional 36 h. Cell viability was measured using the MTT assay. The data represent the mean ± SD of three independent experiments. ## *p* < 0.01 represents significant differences compared to the 5-demethyl NOB-untreated group. ** *p* < 0.01 represents significant differences compared to the Ara C-untreated group.

Cytarabine (Ara-C) is a key therapeutic agent for the standard treatment of AML. We further examined the antileukemic effects of combined 5-demethyl NOB and Ara-C on AML cell lines. THP-1 cells were incubated with Ara-C (10 μM), 5-demethyl NOB (20 and 40 μM) or both compounds, and cell viability was analyzed using the MTT assay. As shown in Figure 8a, cells were treated with Ara-C (0–20 μM) for 48 h, and cell viability was reduced from 100.0 ± 4.2% to 54.3 ± 7.1% in a dose-dependent manner. These data were consistent with our previous report [15]. As shown in Figure 8b, in the Ara-C (10 μM) and 5-demethyl NOB (20 or 40 μM) cotreated groups, a significant reduction in cell viability was noted compared with Ara-C- or 5-demethyl NOB-treated cells (*p* < 0.01). This data indicated that a combination of cytarabine and 5-demethyl NOB demonstrated an enhanced cytotoxic effect for the alleviation of the cell viability compared with cytarabine- or 5-demethyl NOB-alone treated cells. The combination index (CI) values calculated in the combination of Ara-C (10 μM) with 5-demethyl NOB (20 and 40 μM) were 0.72 and 0.91 (CI < 1), respectively. These results demonstrated a synergistic effect of the combination of Ara-C with 5-demethyl NOB treatment in THP-1 cells. A similar synergistic effect of Ara-C (0.125 μM) and 5-demethyl NOB (20 and 40 μM) cotreatment on the reduction of cell viability was also found in U-937 cells (Figure 8c,d). The data demonstrated the therapeutic potential of 5-demethyl NOB supplemented with Ara-C in AML treatment. Our findings suggested that low and nontoxic concentration of 5-demethyl NOB combined with reduced doses of cytarabine treatment resulted in more inhibitory effects on leukemia cell growth.

## 3. Discussion

**Figure 9:**
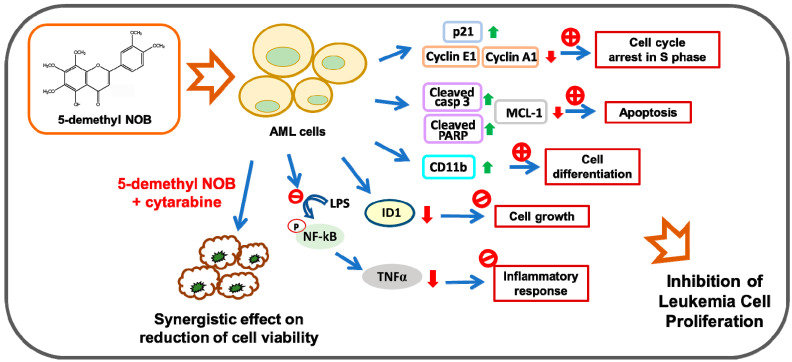
A hypothetical mechanism of the antileukemic effects of 5-demethyl NOB in human AML cells. 5-Demethyl NOB inhibits leukemia cell proliferation through the regulation of cell cycle arrest, apoptosis, cell differentiation, cell growth, and the NF-κB/TNF-α-mediated inflammatory response. 5-Demethyl NOB regulates cell proliferation through a substantial reduction in ID1 expression in AML cells. Cytarabine combined with 5-demethyl NOB showed a synergistic effect on the reduction of cell viability in AML cells.

Citrus PMFs have been reported as safe phytochemicals with limited toxicity and have anticancer applications that are under investigation in animal models and clinical practice. To our knowledge, this is the first report to demonstrate that dietary citrus 5-demethyl NOB inhibits leukemia cell proliferation through the regulation of cell growth, cell cycle distribution, apoptosis, and differentiation in AML cells. We also found that 5-demethyl NOB regulates AML cell proliferation through a substantial decline in ID1 expression and modulation of the NF-κB/TNF-α inflammatory pathway. Additionally, we demonstrated that cytarabine combined with 5-demethyl NOB showed a synergistic effect on decreases in cell viability in leukemia cells (Figure 9). Our findings suggest that 5-demethyl NOB represents a novel agent for AML chemotherapy. Moreover, 5-demethyl NOB has the potential to be used in combination therapy with cytarabine for patients with AML.

5-Demethyl NOB, a flavonoid phytochemical in citrus fruit peel, plays an important role in cancer chemoprevention [17]. In this study, we demonstrated that 5-demethyl NOB (20–80 μM) dramatically reduced AML and CML cell viability. We found that no cytotoxicity was induced by 5-demethyl NOB in normal PBMCs. 5-Demethyl NOB has been reported to inhibit the growth of human solid tumor cells by inducing cell cycle arrest and apoptosis and regulating signaling proteins related to cell proliferation [43,44]. In this study, we found that 5-demethyl NOB inhibited cell growth and induced S phase arrest in THP-1 cells. Cell cycle arrest at the G1/S transition or S phase is associated with increased cyclin-dependent kinase (CDK) inhibitors p21 and reduced levels of cyclin A and E proteins [45]. We found that 5-demethyl NOB increased p21 levels and reduced cyclin E1 and A1 protein levels to impede S phase progression. We further demonstrated that 5-demethyl NOB reduced cell viability by facilitating the cell apoptotic process in leukemia cells. Our findings demonstrated that 5-demethyl NOB possessed antileukemic effects on the inhibition of AML cell proliferation. 5-Demethyl NOB have been reported to induce cytotoxicity in several types of cancer cells. In in vitro assay studies, 5-demethyl NOB significantly reduced the viability of cancer cell lines, including HCT116 (IC_50_ 8.7 and 13.5 μM), CL1-5 (IC_50_ 12.8 μM), CL13 (IC_50_ 21.8 μM) and SH-SY5Y (IC_50_ 32.5 μM), respectively [18,26,44,46,47]. Our previous study showed that NOB inhibited human AML cell proliferation [15]. The IC_50_ values in NOB- and 5-demethyl NOB-treated THP-1 cells were 54.8 μM and 32.3 μM, respectively. These data suggested that 5-demethyl NOB exhibits more inhibitory activities of cell proliferation than NOB in leukemia cells. Similar results indicating that 5-demethyl NOB-induced more anti-proliferative effects than NOB have also been reported in other cells [18,24], compared with its PMF counterpart, suggesting an essential role of the hydroxyl group at the 5-position in the growth inhibition of cancer cells. In this study, we investigated the antileukemic effects of 5-demethyl NOB on leukemia cell lines in vitro. We demonstrated that 5-demethyl NOB significantly inhibited AML cell growth, regulated gene expression or signaling pathways, and enhanced the Ara-C chemotoxicity at concentrations 20–40 μM, which are used in several anticancer studies in vitro, though the dosage is higher than what can be reached in vivo. 5-Demethyl NOB is a PMF, like other flavonoids, and possesses a poor solubility and bioavailability in vivo. To improve the bioactivity of 5-demethyl NOB in vivo, novel systems for delivery or chemical modification may enhance its solubility and achieve the concentration used in this study [48,49,50,51].

Clinical investigations have led to the development of new agents to target AML cell proliferation and survival pathways. In this study, transcriptomic data demonstrated the signaling molecules that respond to 5-demethyl NOB to impede leukemia progression. Using pathway enrichment, REVIGO and GSEA analyses of microarray data, 5-demethyl NOB was found to dramatically downregulate the expression of a gene set involved in the GO BP cell population proliferation in AML cells. These data showed that the differential mRNA expression of 72 genes was markedly downregulated by 5-demethyl NOB, which is involved in the modulation of cell proliferation. Among these genes, we discovered that the mRNA level of the *ID1* gene was the most significantly decreased by 5-demethyl NOB treatment. ID1 protein, a member of the helix-loop-helix (HLH) protein superfamily, mediates dimerization with basic HLH proteins to inhibit the differentiation of progenitor cells, facilitate cell cycle progression, and impede cell senescence [52]. The overexpression or deregulation of the *ID1* gene has been reported to promote tumor development and progression in numerous types of cancers [53]. The downregulation of *ID1* gene expression using antisense oligonucleotides or compound inhibitors alleviates cell proliferation, promotes cell differentiation, and suppresses invasiveness in cancer. Thus, targeting ID1 represents a promising strategy for antitumor therapy [35,54]. Herein, the analysis of data from microarray studies, transcriptional activity of the promoter, real-time PCR, and Western blot revealed that 5-demethyl NOB functions as a strong agent for reduction of both ID1 mRNA and protein levels in THP-1 cells. We demonstrated that ID1 overexpression alone significantly enhanced THP-1 cell proliferation. In 5-demethyl NOB-treated cells, ID1 protein overexpression increased cell viability. These data suggested that the ID1 protein is a critical target for the antiproliferative effect of 5-demethyl NOB in AML cells. In AML, ID1 is highly expressed in leukemia cells and is associated with poor prognosis in patients [55]. ID1 inhibits the expression of p21, which leads to the modulation of the levels of cyclin A and E and promotion of cell cycle progression for leukemogenesis. Additionally, ID1 contributes to tumorigenesis by suppressing cell differentiation [56,57]. In this study, 5-demethyl NOB increased p21 levels and induced cell cycle arrest at S phase. Moreover, 5-demethyl NOB also induced the expression of the CD11b differentiation marker in THP-1, U937, and HL-60 cell lines and indicated that 5-demethyl NOB promoted cell differentiation in these myeloid leukemia cells. ID1 downregulation mediated by 5-demethyl NOB seems to play a critical role in the regulation of cell growth, the cell cycle, and cell differentiation in AML cells. Our results suggest a novel mechanism of the antileukemic activity of 5-demethyl NOB through targeting ID1 expression. These findings support 5-demethyl NOB as having potential utility in the chemotherapy of AML with aberrant ID1 expression. 

Cytokine networks in the inflammatory response exert profound effects on AML progression. The dysregulation of the pro- and anti-inflammatory cytokines in AML may produce a microenvironment for leukemic cell proliferation and survival [8,9]. Several studies have suggested that the NF-κB signaling pathway plays an important role in the progression of AML. NF-κB is a transcription factor that serves as a critical regulator of cell proliferation, survival, and differentiation. NF-κB is activated by the TNF-α signaling pathway and also induces TNF-α expression [58]. High concentrations of TNF-α have been reported to be involved in leukemogenesis, the tumor microenvironment, leukemia cell proliferation, and chemoresistance [41]. Thus, NF-κB and TNF-α may represent potent targets for the intervention of AML. In this study, GSEA data showed that 5-demethyl NOB downregulated genes involved in the NF-κB-mediated TNF-α and inflammatory response pathways. Moreover, 5-demethyl NOB markedly mitigated inflammation-induced NF-κB activation and TNF-α expression in THP-1 cells. These data showed that the attenuation of the NF-κB-activated inflammatory response may be involved in the antiproliferative effect of 5-demethyl NOB in AML cells. More specific inhibitors targeting NF-κB and TNF-α are currently in preclinical development. The natural phytochemical parthenolide has also been shown to inhibit NF-κB signaling and was found to target AML stem and progenitor cell populations [59,60]. Herein, 5-demethyl NOB could regulate LPS-induced NF-κB activation and TNF-α production. Our findings suggest that 5-demethyl NOB is a potential agent for AML treatment through the regulation of the NF-κB signaling pathway and inflammatory cytokine networks.

Cytarabine (Ara-C) inhibits cellular proliferation and promotes cancer cell apoptosis in the treatment of patients with AML. However, chemoresistance to cytarabine remains a common and serious problem, and some elderly patients may not tolerate high doses of Ara-C due to high toxicity in AML therapy [61]. In clinical studies, it is likely that novel agents for molecular targets combined with traditional chemotherapy will achieve the highest response effects for cancer treatment. In this study, we found that drug combination analysis showed synergistic effects on the reduction of cell viability in AML cells treated with cytarabine and 5-demethyl NOB. These data suggested that 5-demethyl NOB has great potential when administered in combination with cytarabine to achieve a synergistic therapeutic response in patients with AML. In lung cancer studies, treatment with the combination of paclitaxel and 5-demethyl NOB showed significantly synergistic antiproliferative effects on cancer cells in vitro and suppressed tumor growth in vivo in mouse models [46]. Our current findings support that 5-demethyl NOB may serve as an effective agent to combine with traditional chemotherapeutic drugs to prevent cancer cell proliferation. We realized that this study exclusively focused on the in vitro effect of 5-demethyl NOB against cell proliferation in AML cell lines. The AML cell lines used in this study may reflect a slight difference from clinical AML, which are heterogenous types. The antileukemic effects of 5-demethyl NOB on AML in animal and human studies warrant further investigation. Furthermore, if applied in a clinical setting the methods of delivering this compound may need more pharmacokinetic and pharmacodynamic studies.

## 4. Materials and Methods 

### 4.1. Chemicals

5-Demethylnobiletin (5-hydroxy-6,7,8,3′,4′-pentamethoxyflavone, 5-demethyl NOB) was isolated as previously described [62]. 3-(4,5-Dimethylthiazol-2-yl)-2,5-diphenyl tetrazolium bromide (MTT), dimethyl sulfoxide (DMSO), RPMI-1640 medium, nonessential amino acids (NEAAs), cytarabine, lipopolysaccharide (LPS), and other chemicals were purchased from Sigma-Aldrich Co. (St. Louis, MO, USA) unless otherwise indicated.

### 4.2. Cell Culture

THP-1, HL-60, HEL 92.1.7, U-937, and K562 cell lines were obtained from Bioresource Collection and Research Center (BCRC, Hsinchu, Taiwan). Peripheral blood mononuclear cells (PBMCs) were isolated and prepared from healthy volunteer donors using Ficoll-Paque Plus Reagent (GE Healthcare, Buckinghamshire, UK). These cells were cultured in RPMI-1640 medium supplemented with 10% fetal bovine serum (FBS) (Thermo Fisher Scientific, Inc., Rockford, IL, USA) and 1% NEAA in a 5% CO_2_ incubator at 37 °C.

### 4.3. Analysis of Cell Viability

Cell viability was determined by MTT assay as previously described [16]. Briefly, cells were treated with vehicle (0.1% DMSO) or 5-demethyl NOB at the indicated concentration for 48 h and incubated with 1 mg/mL MTT reagent for 3 h at 37 °C. The cell pellet was collected and dissolved in 0.5 mL DMSO followed by the detection of the absorbance at 550 nm as cell viability.

### 4.4. Flow Cytometric Analysis of Cell Cycle Progression and Apoptotic Cells

Standard flow cytometric analysis was used to detect cell cycle distribution and apoptotic cells as previously described [15,16]. Briefly, for the detection of cell cycle progression, the cells were treated with vehicle or 5-demethyl NOB for 24–72 h, incubated with PBS containing 70% ethanol, and stored at −20 °C for 24 h. The cells were stained with propidium iodide (PI) buffer (PBS containing 20 μg/mL PI, 200 μg/mL RNaseA and 0.1% Triton X-100) in the dark at room temperature for 30 min. Cell cycle distribution was measured on a Gallios Flow Cytometer using Kaluza analysis software (Beckman Coulter). An Annexin V-FITC Apoptosis Detection Kit (Strong Biotech, Taipei, Taiwan) was used to assess the apoptotic cell population according to the manufacturer’s instructions. Cells were washed in ice-cold PBS, resuspended in buffer containing Annexin V-FITC and PI, and incubated in the dark at room temperature for 15 min. Apoptotic cells were detected by flow cytometric analysis. The apoptotic cell population was detected on a FACSCalibur and analyzed using Cell Quest Pro software (BD Biosciences, San Jose, CA, USA).

### 4.5. Western Blot Analysis

Cells were treated with vehicle or 5-demethyl NOB (20 and 40 μM) for 24 or 48 h. Total cellular proteins were extracted using RIPA buffer (Thermo Fisher Scientific), separated by 10% or 12% SDS–PAGE, and then transferred onto a PVDF membrane (PerkinElmer, Boston, MA, USA). The membranes were incubated with specific antibodies for human proteins: p21, CDK2, cyclin E1, cleaved-caspase 3, PARP1, p-p65, p65, and TNF-α (Cell Signaling Technology, Danvers, MA, USA); cyclin A1 (Abcam, Cambridge, MA, USA); Mcl-1, Bcl-2, caspase 3, and ID1 (GeneTex, Irvine, CA, USA); β-actin (Sigma–Aldrich) and actin (Thermo Fisher Scientific). The blots were incubated with HRP-conjugated secondary antibodies (Santa Cruz Biotechnology), and proteins were detected using Amersham ECL™ Prime Western Blotting Detection Reagent. The signal was visualized on Amersham Hyperfilm™ ECL (GE Healthcare, Buckinghamshire, UK).

### 4.6. Reverse-Transcription Quantitative Polymerase Chain Reaction (RT–qPCR) Analysis 

RNA was prepared from cells using a blood/cultured cell total RNA purification mini kit (FAVORGEN Biotech, Ping-Tung, Taiwan) according to the manufacturer’s instructions followed by cDNA synthesis using the High-Capacity cDNA Reverse Transcription kit (Applied Biosystem). Quantitative real-time PCR was performed in a reaction mixture that contained cDNA, specific primers (Table 2) and Maxima^TM^ SYBR Green/ROX qPCR Master Mix (Thermo Fisher Scientific). Real-time PCR amplification was performed using a Roche LightCycler 480 Real-Time PCR System (Roche Diagnostics, Rotkreuz, Switzerland). The ∆∆C_t_ method was used for data analysis, and gene expression was estimated in triplicate samples and normalized to the GAPDH level.

### 4.7. RNA Preparation and cDNA Microarray Analysis

RNA was isolated from compound-treated cells for cDNA microarray analysis as previously described [16]. Briefly, cellular RNA was prepared from 48 h of vehicle- or 5-demethyl NOB-treated THP-1 cells using the Illustra RNA Spin Mini RNA Isolation Kit (GE Healthcare) according to the manufacturer’s instructions. Fluorescence-labeled antisense RNA (aRNA) targets were prepared using an Onearray Amino Allyl aRNA Amplification Kit (Phalanx Biotech Group, Hsinchu, Taiwan) and Cy5 dyes (Amersham Pharmacia, Piscataway, NJ, USA). Fluorescent targets were hybridized with Human Whole Genome One Array Plus Version 7.1 (HOA 7.1, Phalanx) using the Phalanx OneArray Plus Protocol. The signals were scanned using an Agilent G2505C scanner based on the Agilent 0.1 XDR Protocol (Agilent Technologies, Santa Clara, CA, USA). Data were analyzed and processed using GenePix 4.1 software (Molecular Devices, Sunnyvale, CA, USA) and Rosetta Resolver 7.2 System (Rosetta Biosoftware, Seattle, WA, USA). The normalized intensities of each spot were transformed to the log_2_ (fold change) of gene expression. Differentially expressed genes (DEGs) associated with the treatment of 5-demethyl NOB for 48 h at |log_2_ (fold change)| ≥ 1.0 or | log_2_ (fold change)| ≥ 0.5 and *p* value < 0.05 were selected for further analysis.

### 4.8. Gene Ontology and Gene Set Enrichment Analysis (GSEA)

Significant DEGs (*p* < 0.05) in the cDNA microarray were analyzed by Gene Ontology (GO) term enrichment to determine the biological processes (BPs) in 5-demethyl NOB-treated cells [63]. The “Reduce plus Visualize Gene Ontology” (REVIGO) web-based tool (http://revigo.irb.hr/ (accessed on 20 February 2022)) was used to summarize nonredundant GO terms [38]. Gene set enrichment analysis (GSEA) [64] (http://software.broadinstitute.org/gsea/ (accessed on 1 March 2022)) was performed to analyze the statistically significant GO BPs and the particular hallmark in which the 5-demethyl NOB-treated cells are involved [65]. 

### 4.9. Preparation of ID1 Promoter-Reporter Constructs, Plasmid Transfection, and Luciferase Reporter Assay

The promoter DNA of human ID1 gene from nucleotides −1362 to +1 was PCR-amplified using primers (Hu-ID1-pro-F: 5′-TGAATTCACTCAGCTGCAGAG-3′ and Hu-ID1-pro-R: 5′-TGATTCTTGGCGACTGGCTG-3′) [66] and human genomic DNA (Promega Corporation, Madison, WI, USA) as a template. The DNA fragment was inserted into the pGL4.17[luc2/Neo] vector (Promega) to produce an ID1 promoter-luciferase reporter plasmid (pGL4.17-ID1-P1). For reporter plasmid transfection, THP-1 cells (8 × 10^5^/well) were seeded in six-well plates and cotransfected with pGL4.17-ID1-P1 and pRL *Renilla* Luciferase Control Reporter Vector (pRL-CMV Vector) using the TransIT-X2 Dynamic Delivery System (Mirus Bio, Madison, WI, USA). After transfection, the cells were treated with vehicle or 5-demethyl NOB (20 and 40 μM) for an additional 24 h. The protein lysates were prepared using Passive Lysis Buffer, and luciferase activities were determined by the Dual-Luciferase Reporter Assay System Kit (Promega). The intensities of the luciferase activity measured in the lysates of the pGL4.17-ID1-P1-transfected cells were normalized to the activity of the Renilla luciferase control. For the overexpression of ID1 protein, THP-1 cells were transfected with pCMV6 control vector or pCMV6-ID1 expression plasmids (OriGene Technologies, Rockville, MD, USA) using the TransIT-X2 Dynamic Delivery System for 24 h. The plasmid-transfected cells were treated with vehicle or 5-demethyl NOB for an additional 48 h, and cell viability was measured by MTT assay.

### 4.10. Combination Analysis

To evaluate the pharmacological interactions of the combination of cytarabine and 5-demethyl NOB, the combination index (CI) was calculated, where CI < 1, =1, and >1 indicates synergism, additive effect, and antagonism, respectively [67]. The CI was calculated as follows: CI = (C_cytarabine_)/(C_x_)_cytarabine_ + (C_5-demethyl NOB_)/(C_x_)_5-demethyl NOB_, where (C_x_)_cytarabine_ and (C_x_)_5-demethyl NOB_ are the doses of cytarabine and 5-demethyl NOB, respectively, alone inhibiting x%, and (C_cytarabine_) and (C_5-demethyl NOB_) are the doses of cytarabine and 5-demethyl NOB, respectively, in combination, which gives the experimentally observed x% inhibition [16].

### 4.11. Statistical Analysis

All experiments were performed in at least three independent experiments, and each experiment was repeated thrice. The data are expressed as the mean ± SD. Statistical analyses were performed using Student’s *t* test for two-group comparisons. The results from comparisons with multiple groups were analyzed using one-way ANOVA with Dunnett’s post hoc test, and a *p* value < 0.05 was considered significant.

## 5. Conclusions

In this study, for the first time, we demonstrated that 5-demethyl NOB inhibits cancer cell proliferation, suppresses the gene expression of ID1, regulates the NF-κB pathway, and exerts antileukemic effects in human AML cells. 5-Demethyl NOB also sensitized AML cells to the clinical chemotherapeutic cytarabine. Our current findings demonstrate that citrus 5-demethyl NOB may serve as a potential phytochemical for chemotherapy of human hematological malignancies.

## Figures and Tables

**Table 1:** The differentially expressed genes (DEGs) of 5-demethyl NOB-treated THP-1 cells.

Differentially Expressed Genes (DEGs)	5-Demethyl NOB Treatment 48 h versus 0 h
Genes in upregulated expression	552
Genes in downregulated expression	694

**Table 2:** The primer pairs used in real-time PCR.

Genes	Primers
CD11b	5′-ACTTGCAGTGAGAACACGTATG-3′ 5′-AGAGCCATCAATCAAGAAGGC-3′
INHBE	5′-GGTCTGTGTGTCCCTCCTGT-3′ 5′-GGAGCTGTAGGCTGAAGTGG-3′
ID1	5′-CGGATCGAGGGAGAACAAG-3′ 5′-TCCCACCCCCTAAAGTCTCT-3′
GAPDH	5′-CATGAGAAGTATGACAACAGCCT-3′ 5′-AGTCCTTCCACGATACCAAAGT-3′

## Data Availability

Not applicable.

## Supplementary Materials

The following supporting information can be downloaded at: https://www.mdpi.com/article/10.3390/ijms23137392/s1.
