# Supplementary material for: 5-Demethylnobiletin Inhibits Cell Proliferation, Downregulates ID1 Expression, Modulates the NF-κB/TNF-α Pathway and Exerts Antileukemic Effects in AML Cells
Source: Int J Mol Sci. 2022 Jul 2;23(13):7392. doi: 10.3390/ijms23137392 (PMC9266321; doi:10.3390/ijms23137392)
Supplement: Supplementary file 1 [file ijms-23-07392-s001.zip › ijms-1786947-supplementary.pdf]

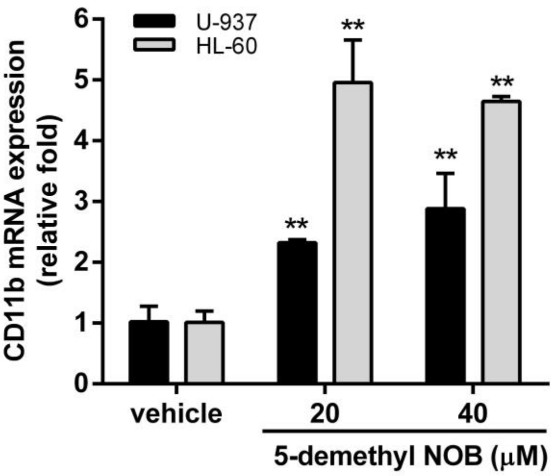

Figure S1. 5-Demethyl NOB induced cell differentiation in U-937 and HL-60 cells. U-937 and HL-60 cells were treated with vehicle (0.1% DMSO) or 5-demethyl NOB (20-100 μM) for 48 h. The level of CD11b mRNA was measured by RT-qPCR analysis. The data represent the mean ± SD of three independent experiments. **p<0.01 represents significant differences compared to the vehicle-treated group.

Table S1. Biological processes in the Gene Ontology (GO) analysis of up- and downregulated genes, associated with DEGs of 5-demethyl NOB-treated THP-1 cells.

GO ID	Description	Genes in term	P value	p. adjust	Count
GO:0009987	cellular process	15796	0.00333839	0.08201	822
GO:0065007	biological regulation	12054	0.00011811	0.008583	659
GO:0050789	regulation of biological process	11437	0.00017309	0.011379	628
GO:0008152	metabolic process	11555	0.00824039	0.140015	618
GO:0071704	organic substance metabolic process	11077	0.00199881	0.060252	601
GO:0050794	regulation of cellular process	10788	0.00010994	0.008184	598
GO:0044237	cellular metabolic process	10730	0.00096117	0.037275	587
GO:0044238	primary metabolic process	10696	0.00167481	0.052791	583
GO:0006807	nitrogen compound metabolic process	10182	0.00190251	0.058629	557
GO:0043170	macromolecule metabolic process	9451	0.00108518	0.039846	523
GO:0050896	response to stimulus	8776	0.00612902	0.120537	481
GO:0051716	cellular response to stimulus	7299	0.00084881	0.033869	415
GO:0019222	regulation of metabolic process	6765	1.722E-06	0.000865	409
GO:0032501	multicellular organismal process	7363	0.01896676	0.212545	403
GO:0009058	biosynthetic process	6546	2.9282E-06	0.000872	396
GO:0034641	cellular nitrogen compound metabolic process	6688	9.4535E-05	0.007332	392
GO:1901576	organic substance biosynthetic process	6475	4.4789E-06	0.001066	391
GO:0044249	cellular biosynthetic process	6378	4.3727E-06	0.001066	386
GO:1901360	organic cyclic compound metabolic process	6286	7.2901E-05	0.00637	372
GO:0006725	cellular aromatic compound metabolic process	6071	3.7838E-05	0.004146	363
GO:0023052	signaling	6496	0.01014626	0.15599	362
GO:0007154	cell communication	6516	0.01228817	0.175259	362
GO:0046483	heterocycle metabolic	6032	3.7317E-05	0.004146	361

	process				
GO:0032502	developmental process	6099	0.00030509	0.017405	357
GO:0048518	positive regulation of biological process	5680	2.3981E-06	0.000872	351
GO:0048856	anatomical structure development	5666	6.2333E-05	0.006048	340
GO:0048519	negative regulation of biological process	5154	1.0749E-08	7.42E-05	337
GO:0007275	multicellular organism development	5190	0.00014024	0.009946	312
GO:2000112	regulation of cellular macromolecule biosynthetic process	4243	3.2244E-05	0.003905	266
GO:0042221	response to chemical	4373	0.00364282	0.086602	256
GO:0032774	RNA biosynthetic process	3857	1.8006E-06	0.000865	253
GO:0006351	transcription, DNA-templated	3823	1.2813E-06	0.000865	252
GO:0097659	nucleic acid-templated transcription	3841	1.8796E-06	0.000865	252
GO:0048583	regulation of response to stimulus	4043	0.00016188	0.010952	250
GO:0048869	cellular developmental process	4207	0.00235576	0.066375	249
GO:2001141	regulation of RNA biosynthetic process	3690	7.2574E-06	0.001392	240
GO:0006355	regulation of transcription, DNA-templated	3659	5.5756E-06	0.001113	239
GO:1903506	regulation of nucleic acid-templated transcription	3682	8.8807E-06	0.00146	239
GO:0030154	cell differentiation	4036	0.00291194	0.07643	239
GO:0006950	response to stress	3854	0.00242451	0.066787	230
GO:0006464	cellular protein modification process	4108	0.04098956	0.308212	230
GO:0023051	regulation of signaling	3432	5.402E-05	0.005367	220
GO:0065008	regulation of biological quality	3551	0.00132138	0.044934	216
GO:0070887	cellular response to	2980	5.4047E-06	0.001113	201

	chemical stimulus				
GO:0065009	regulation of molecular function	3359	0.00558741	0.116925	200
GO:0010033	response to organic substance	3051	7.1961E-05	0.00637	198
GO:0051239	regulation of multicellular organismal process	2864	2.8358E-06	0.000872	196
GO:0002376	immune system process	2928	5.2793E-05	0.005359	192
GO:0006793	phosphorus metabolic process	3309	0.02273925	0.234658	191
GO:0050793	regulation of developmental process	2442	2.5532E-07	0.000865	177
GO:0032879	regulation of localization	2641	0.00020558	0.012785	172
GO:0009653	anatomical structure morphogenesis	2503	2.4323E-05	0.003109	170
GO:0009605	response to external stimulus	2238	9.5323E-06	0.00153	157
GO:0006366	transcription from RNA polymerase II promoter	2169	2.196E-05	0.002972	151
GO:0051704	multi-organism process	2466	0.00829845	0.140059	150
GO:0006928	movement of cell or subcellular component	2011	1.3191E-06	0.000865	148
GO:0008219	cell death	2160	8.852E-05	0.007105	147
GO:0016310	phosphorylation	2316	0.00784535	0.134383	142
GO:0012501	programmed cell death	2031	0.00011025	0.008184	139
GO:0006357	regulation of transcription from RNA polymerase II promoter	1978	7.2054E-05	0.00637	137
GO:0009888	tissue development	1933	3.6836E-05	0.004146	136
GO:0006468	protein phosphorylation	1924	0.00067589	0.028624	128
GO:0040011	locomotion	1762	3.5905E-05	0.004131	126
GO:0008283	cell proliferation	2048	0.01099745	0.166847	126
GO:0006955	immune response	2071	0.02511243	0.245708	124
GO:0031399	regulation of protein modification process	1763	0.00042735	0.020774	120
GO:0006952	defense response	1651	4.3228E-05	0.004663	119
GO:0048870	cell motility	1529	5.5445E-06	0.001113	116
GO:0051674	localization of cell	1529	5.5445E-06	0.001113	116

GO:0042981	regulation of apoptotic process	1507	7.7082E-06	0.001402	114
GO:0019220	regulation of phosphate metabolic process	1740	0.00318326	0.079906	113
GO:0042592	homeostatic process	1683	0.0103096	0.158149	106
GO:0044093	positive regulation of molecular function	1771	0.03733337	0.294399	106
GO:0042325	regulation of phosphorylation	1502	0.00127624	0.044049	102
GO:0002682	regulation of immune system process	1525	0.00207315	0.061952	102
GO:2000113	negative regulation of cellular macromolecule biosynthetic process	1583	0.00631033	0.121338	102
GO:1901700	response to oxygen-containing compound	1535	0.00358557	0.085942	101
GO:0007267	cell-cell signaling	1583	0.01164082	0.170971	100

Table S2. Z score-elite to perform GO pruning for over-representation analysis (ORA) to determine BPs are enriched in up-regulation of gene sets of 5-demethyl NOB-treated cells.

**Ontology Name (Ontology-ID)**	**Number in Ontology**	**Z Score**	**FisherExactP**	**AdjustedP**
regulation of melanocyte differentiation (GO:0045634)	6	8.57748987	0.000135088	0.303291312
base conversion or substitution editing (GO:0016553)	7	7.888479254	0.00023303	0.319354505
response to nicotine (GO:0035094)	38	7.413373511	6.95E-06	0.053629855
L-serine metabolic process (GO:0006563)	8	7.329666839	0.000367531	0.362123628
neurexin binding (GO:0042043)	13	5.556348362	0.001747336	0.653836658
serine family amino acid biosynthetic process (GO:0009070)	14	5.316930668	0.002192355	0.68910097
developmental process (GO:0032502)	4327	4.704277813	6.75E-06	0.053629855
locomotory behavior (GO:0007626)	158	4.630830505	0.000248982	0.319354505
regulation of metabolic process (GO:0019222)	5797	4.567254189	1.02E-05	0.053629855
neuron-neuron synaptic transmission (GO:0007270)	44	4.565548548	0.001501427	0.620128701
cellular response to hypoxia (GO:0071456)	80	4.455814687	0.000876245	0.535389999
reactive oxygen species metabolic process (GO:0072593)	81	4.412745072	0.000943573	0.535389999
peptidyl-threonine phosphorylation (GO:0018107)	32	4.360884349	0.003160751	0.856454641
hydrolase activity, acting on carbon-nitrogen (but not peptide) bonds, in cyclic amidines (GO:0016814)	33	4.270015079	0.003542919	0.871583442
peptidyl-serine phosphorylation (GO:0018105)	70	4.058041512	0.002261814	0.68917197
nuclear inner membrane (GO:0005637)	36	4.018462464	0.004874942	0.910092474
carboxylic acid transport (GO:0046942)	196	3.779786557	0.001499609	0.620128701
Rho guanyl-nucleotide exchange factor activity (GO:0005089)	77	3.757954887	0.00365685	0.871583442
adult behavior (GO:0030534)	122	3.739220884	0.002495858	0.740092592
defense response to virus (GO:0051607)	151	3.626587082	0.002634963	0.766871877

phospholipid binding (GO:0005543)	537	3.399391269	0.002091882	0.68910097
fibroblast growth factor receptor signaling pathway (GO:0008543)	165	3.317303207	0.004735488	0.910092474
nerve growth factor receptor signaling pathway (GO:0048011)	284	3.28090124	0.003556264	0.871583442
regulation of signaling (GO:0023051)	2072	3.231634987	0.002280284	0.68917197
negative regulation of biological process (GO:0048519)	3354	3.229507585	0.001939408	0.68910097

Table S3. Z score-elite to perform GO pruning for ORA to determine BPs are enriched in down-regulation of gene sets of 5-demethyl NOB-treated cells.

**Ontology Name (Ontology-ID)**	**Number in Ontology**	**Z Score**	**FisherExactP**	**AdjustedP**
macromolecule biosynthetic process (GO:0009059)	3076	7.360361121	1.41E-11	2.22E-07
response to wounding (GO:0009611)	577	7.279810172	2.08E-09	1.92E-06
regulation of response to external stimulus (GO:0032101)	425	7.230007024	6.34E-09	4.75E-06
RNA biosynthetic process (GO:0032774)	2587	6.881282891	2.79E-10	5.48E-07
regulation of biosynthetic process (GO:0009889)	4056	6.764983199	2.47E-10	5.48E-07
nucleic acid binding (GO:0003676)	3801	6.643755478	4.48E-10	7.83E-07
regulation of nitrogen compound metabolic process (GO:0051171)	4215	6.491559476	9.79E-10	1.28E-06
RAGE receptor binding (GO:0050786)	9	6.366768896	0.000806269	0.081750518
muscle organ morphogenesis (GO:0048644)	9	6.366768896	0.000806269	0.081750518
negative regulation of biological process (GO:0048519)	3354	6.318352382	3.54E-09	2.93E-06
organ development (GO:0048513)	1122	5.920573148	1.63E-07	7.32E-05
neutrophil chemotaxis (GO:0030593)	37	5.815355333	0.00014343	0.023980248
Nucleus (GO:0005634)	5712	5.745613228	3.03E-08	1.68E-05
cellular response to zinc ion (GO:0071294)	11	5.66874635	0.001532574	0.123517574
positive regulation of cellular process (GO:0048522)	3483	5.610790188	1.22E-07	5.62E-05
regulation of multicellular organismal development (GO:2000026)	1214	5.49852977	8.54E-07	0.000353262
oocyte development (GO:0048599)	12	5.384222221	0.002010205	0.151886435
positive regulation of developmental process (GO:0051094)	732	5.356725902	3.72E-06	0.001245438
adipose tissue development (GO:0060612)	21	5.264192124	0.001029963	0.093160382
Intracellular (GO:0005622)	1679	5.211591409	2.09E-06	0.000782461
digestive tract morphogenesis (GO:0048546)	22	5.111282344	0.001237136	0.106960453
DNA-dependent DNA replication initiation (GO:0006270)	22	5.111282344	0.001237136	0.106960453

response to organic cyclic compound (GO:0014070)	224	5.068078439	3.95E-05	0.008742307
sequence-specific DNA binding transcription factor activity (GO:0003700)	1105	4.988714918	7.05E-06	0.002172239
response to corticosteroid stimulus (GO:0031960)	128	4.944416466	0.000125764	0.022260492
regulation of myeloid cell apoptosis (GO:0033032)	14	4.904844533	0.003218871	0.202362535
glial cell migration (GO:0008347)	14	4.904844533	0.003218871	0.202362535
homeostasis of number of cells (GO:0048872)	95	4.842104051	0.000247191	0.034998641
regulation of binding (GO:0051098)	194	4.783301497	0.000106436	0.019450634
Nucleosome (GO:0000786)	115	4.759823859	0.000230003	0.033697955
embryonic morphogenesis (GO:0048598)	386	4.728371988	5.10E-05	0.010980407
cellular response to cadmium ion (GO:0071276)	15	4.69989484	0.003958384	0.232996127
purinergic receptor activity (GO:0035586)	39	4.524093727	0.001570667	0.125941824
mammary gland epithelial cell differentiation (GO:0060644)	16	4.51324348	0.004792996	0.257968241
lung morphogenesis (GO:0060425)	16	4.51324348	0.004792996	0.257968241
ribonucleoside catabolic process (GO:0042454)	16	4.51324348	0.004792996	0.257968241
response to interferon-alpha (GO:0035455)	16	4.51324348	0.004792996	0.257968241
heterocycle biosynthetic process (GO:0018130)	254	4.478719897	0.000173029	0.027748239
response to other organism (GO:0051707)	460	4.458508979	0.000126062	0.022260492
zinc ion binding (GO:0008270)	2188	4.441859025	3.90E-05	0.008742307
positive regulation of multicellular organismal process (GO:0051240)	561	4.273922072	0.000178998	0.02841555
cardiac septum morphogenesis (GO:0060411)	43	4.217344032	0.002443836	0.176180387
osteoblast differentiation (GO:0001649)	59	4.177375884	0.0018505	0.143972597
placenta blood vessel development (GO:0060674)	31	4.06411586	0.004553087	0.256014421
transcription factor complex (GO:0005667)	357	4.053902256	0.000387864	0.04880824
extracellular region part (GO:0044421)	1294	4.014004551	0.000254325	0.035687279
nucleosome assembly (GO:0006334)	164	3.948564026	0.001049211	0.094225136
transmembrane receptor protein	187	3.947020246	0.000930272	0.088073262

serine/threonine kinase signaling pathway (GO:0007178)				
multicellular organismal process (GO:0032501)	3075	3.902724402	0.000184334	0.028969939
biomineral tissue development (GO:0031214)	67	3.773950895	0.003538272	0.216715997
cytokine activity (GO:0005125)	249	3.701696961	0.0013129	0.111532615
translational termination (GO:0006415)	108	3.697086634	0.00267975	0.182585571
transcription factor binding (GO:0008134)	502	3.679733759	0.000969317	0.090677308
cell activation (GO:0001775)	594	3.667082356	0.000913158	0.087798771
system development (GO:0048731)	787	3.635033067	0.001083921	0.096789179
regulation of cell-substrate adhesion (GO:0010810)	110	3.634815854	0.003002955	0.191847329
regulation of ossification (GO:0030278)	178	3.63328876	0.002021944	0.15198069
anatomical structure formation involved in morphogenesis (GO:0048646)	660	3.632124683	0.001009588	0.092787588
pigment metabolic process (GO:0042440)	71	3.595116498	0.004718971	0.257968241
response to lipid (GO:0033993)	71	3.595116498	0.004718971	0.257968241
response to drug (GO:0042493)	340	3.546389772	0.002008685	0.151886435
cofactor biosynthetic process (GO:0051188)	159	3.52682017	0.002804565	0.182585571
nucleobase, nucleoside, nucleotide and nucleic acid biosynthetic process (GO:0034654)	186	3.466587856	0.002846365	0.182585571
pattern binding (GO:0001871)	213	3.431979867	0.002789777	0.182585571
structural constituent of ribosome (GO:0003735)	193	3.327775383	0.00377256	0.224581624
positive regulation of protein serine/threonine kinase activity (GO:0071902)	246	3.315665013	0.003297497	0.205648666
G1/S transition of mitotic cell cycle (GO:0000082)	169	3.305842127	0.004331826	0.247559928
regulation of growth (GO:0040008)	572	3.296905376	0.003267568	0.204594032
regulation of caspase activity (GO:0043281)	195	3.289238713	0.004077251	0.236450454
cellular component morphogenesis (GO:0032989)	482	3.26353922	0.003901424	0.230506713
response to oxidative stress (GO:0006979)	254	3.187898147	0.004317985	0.247559928
regulation of cell activation (GO:0050865)	460	3.173132429	0.003554764	0.216715997
cell proliferation (GO:0008283)	621	3.146180856	0.004821799	0.258632761

Table S4. The core enrichment genes associated with cell population proliferation (GO: 0008283) was down-regulated by 5-demethyl NOB treatment.

Symbol	Title	log2(FC)	p value	Rank in metric score
ID1	inhibitor of DNA binding 1, HLH protein	-4.32	1.21E-05	-4.491
PDGFA	platelet derived growth factor subunit A	-2.67	2.44E-05	-3.817
EGR3	early growth response 3	-2.90	2.45E-04	-3.56
TNF	tumor necrosis factor	-2.32	6.02E-06	-3.339
CRIP2	cysteine rich protein 2	-2.87	6.48E-05	-2.969
CDK6	cyclin dependent kinase 6	-1.96	1.17 E-04	-2.957
ATF3	activating transcription factor 3	-1.98	1.13 E-03	-2.771
KLF10	Kruppel like factor 10	-1.76	4.28E-05	-2.719
RGCC	regulator of cell cycle	-2.30	2.32E-04	-2.716
GPR183	G protein-coupled receptor 183	-1.59	1.30E-04	-2.676
PHLDA2	pleckstrin homology like domain family A member 2	-1.71	4.28E-06	-2.665
TNFRSF21	TNF receptor superfamily member 21	-1.64	4.80E-04	-2.585
EGR1	early growth response 1	-1.64	5.98E-05	-2.56
KLF9	Kruppel like factor 9	-1.59	1.21E-04	-2.509
SMAD1	SMAD family member 1	-1.54	1.55E-04	-2.436
ZFP36	ZFP36 ring finger protein	-1.49	9.57E-04	-2.366
PITX2	paired like homeodomain 2	-1.51	7.87E-04	-2.344
TNFSF14	TNF superfamily member 14	-1.57	6.71E-04	-2.287
CYP1B1	cytochrome P450 family 1 subfamily B member 1	-1.41	3.00E-04	-2.255
CXCL10	C-X-C motif chemokine ligand 10	-1.37	7.64E-05	-2.207
VEGFC	vascular endothelial growth factor C	-1.35	0.14E-04	-2.174
OSM	oncostatin M	-1.34	4.48E-05	-2.169
PDPN	podoplanin	-1.33	2.40E-04	-2.157
ANXA1	annexin A1	-1.32	2.51 E-04	-2.146
F3	coagulation factor III, tissue factor	-1.32	3.68E-04	-2.119
CCNE1	cyclin E1	-1.29	7.25E-05	-2.102

PROK2	prokineticin 2	-1.28	7.23E-04	-2.088
TBX2	T-box transcription factor 2	-1.31	1.27 E-03	-2.085
LGR4	leucine rich repeat containing G protein-coupled receptor 4	-1.27	2.18E-05	-2.069
JARID2	jumonji and AT-rich interaction domain containing 2	-1.44	3.68E-03	-2.053
HES1	hes family bHLH transcription factor 1	-1.26	1.73E-04	-2.05
CDCA7	cell division cycle associated 7	-1.31	1.629 E-04	-2.032
TREM2	triggering receptor expressed on myeloid cells 2	-1.24	2.64E-04	-2.027
DIXDC1	DIX domain containing 1	-1.25	7.61E-04	-2.024
NME1	NME/NM23 nucleoside diphosphate kinase 1	-1.05	1.35E-04	-1.996
CRTAM	cytotoxic and regulatory T cell molecule	-1.29	1.49 E-03	-1.99
FGF2	fibroblast growth factor 2	-1.74	3.04E-05	-1.982
ASCL2	achaete-scute family bHLH transcription factor 2	-1.20	4.08 E-04	-1.976
IGFBP7	insulin like growth factor binding protein 7	-1.27	1.07 E-03	-1.965
OSGIN1	oxidative stress induced growth inhibitor 1	-1.18	1.98 E-04	-1.956
PRDX4	peroxiredoxin 4	-1.46	5.53E-03	-1.94
ING2	inhibitor of growth family member 2	-1.17	1.22E-03	-1.939
GAL	galanin and GMAP prepropeptide	-1.42	4.27E-04	-1.936
CXCL11	C-X-C motif chemokine ligand 11	-1.18	1.94E-04	-1.936
GATA2	GATA binding protein 2	-1.15	9.08E-04	-1.914
ISL1	ISL LIM homeobox 1	-1.17	8.30E-04	-1.907
IL18RAP	interleukin 18 receptor accessory protein	-1.16	7.25E-05	-1.9
CSPG4	chondroitin sulfate proteoglycan 4	-1.16	3.54E-03	-1.891
ACVR1C	activin A receptor type 1C	-1.50	8.19E-03	-1.891
CHST11	carbohydrate sulfotransferase 11	-1.13	6.82E-04	-1.87
AHR	aryl hydrocarbon receptor	-1.33	4.00E-04	-1.799
IFIT3	interferon induced protein with tetratricopeptide repeats 3	-1.07	1.48E-04	-1.785

ADORA3	adenosine A3 receptor	-1.07	4.77E-04	-1.785
PSMB10	proteasome 20S subunit beta 10	-1.07	1.73E-03	-1.763
GINS4	GINS complex subunit 4	-1.06	1.613E-03	-1.755
FOXF1	forkhead box F1	-1.06	6.71E-05	-1.754
CCND1	cyclin D1	-1.05	9.65E-04	-1.734
PRTN3	proteinase 3	-1.32	3.47E-04	-1.727
CDKN1A	cyclin dependent kinase inhibitor 1A	-1.03	3.72 E-04	-1.713
JUNB	"JunB proto-oncogene, AP-1 transcription factor subunit	-1.02	2.15E-04	-1.7
SCG2	secretogranin II	-0.98	2.55E-03	-1.665
SETMAR	SET domain and mariner transposase fusion gene	-0.94	1.42E-04	-1.583
LMNA	lamin A/C	-0.94	7.34E-04	-1.581
NRARP	NOTCH regulated ankyrin repeat protein	-0.94	5.90E-04	-1.571
MARCKSL1	MARCKS like 1	-0.92	4.49E-04	-1.552
SIX1	SIX homeobox 1	-1.02	7.00E-03	-1.55
LTK	leukocyte receptor tyrosine kinase	-0.92	1.15E-04	-1.544
IGFBP3	insulin like growth factor binding protein 3	-1.02	5.07E-03	-1.542
PYCARD	PYD and CARD domain containing	-0.91	2.27E-04	-1.534
PNP	purine nucleoside phosphorylase	-0.88	3.55E-03	-1.511
WFDC1	WAP four-disulfide core domain 1	-0.90	2.37E-04	-1.507
LTBP3	latent transforming growth factor beta binding protein 3	-0.88	3.69E-03	-1.502

Table S5. The hallmark gene sets of molecular pathways enriched and down-regulated in response to 5-demethyl NOB treatment.

GENE SET	SIZE	ES	NES	NOM p-value	FDR q-value	LEADING EDGE
HALLMARK TNFA SIGNALING VIA NFKB	82	-0.52	-3.43	0	0	tags=46%, list=12%, signal=51%
HALLMARK INFLAMMATORY RESPONSE	57	-0.4	-2.56	0	0	tags=58%, list=28%, signal=79%
HALLMARK MYC TARGETS V1	59	-0.38	-2.38	0	0	tags=95%, list=57%, signal=215%
HALLMARK TGF BETA SIGNALING	16	-0.59	-2.31	0	0	tags=44%, list=12%, signal=49%
HALLMARK EPITHELIAL MESENCHYMAL TRANSITION	43	-0.39	-2.17	0	0.003	tags=35%, list=13%, signal=40%
HALLMARK ALLOGRAFT REJECTION	36	-0.4	-2.08	0.002	0.008	tags=61%, list=32%, signal=88%
HALLMARK E2F TARGETS	44	-0.33	-1.89	0.003	0.021	tags=77%, list=48%, signal=147%
HALLMARK MYC TARGETS V2	18	-0.48	-1.95	0.004	0.016	tags=94%, list=48%, signal=181%
